# Ensembl 2023

**DOI:** 10.1093/nar/gkac958

**Published:** 2022-11-01

**Authors:** Fergal J Martin, M Ridwan Amode, Alisha Aneja, Olanrewaju Austine-Orimoloye, Andrey G Azov, If Barnes, Arne Becker, Ruth Bennett, Andrew Berry, Jyothish Bhai, Simarpreet Kaur Bhurji, Alexandra Bignell, Sanjay Boddu, Paulo R Branco Lins, Lucy Brooks, Shashank Budhanuru Ramaraju, Mehrnaz Charkhchi, Alexander Cockburn, Luca Da Rin Fiorretto, Claire Davidson, Kamalkumar Dodiya, Sarah Donaldson, Bilal El Houdaigui, Tamara El Naboulsi, Reham Fatima, Carlos Garcia Giron, Thiago Genez, Gurpreet S Ghattaoraya, Jose Gonzalez Martinez, Cristi Guijarro, Matthew Hardy, Zoe Hollis, Thibaut Hourlier, Toby Hunt, Mike Kay, Vinay Kaykala, Tuan Le, Diana Lemos, Diego Marques-Coelho, José Carlos Marugán, Gabriela Alejandra Merino, Louisse Paola Mirabueno, Aleena Mushtaq, Syed Nakib Hossain, Denye N Ogeh, Manoj Pandian Sakthivel, Anne Parker, Malcolm Perry, Ivana Piližota, Irina Prosovetskaia, José G Pérez-Silva, Ahamed Imran Abdul Salam, Nuno Saraiva-Agostinho, Helen Schuilenburg, Dan Sheppard, Swati Sinha, Botond Sipos, William Stark, Emily Steed, Ranjit Sukumaran, Dulika Sumathipala, Marie-Marthe Suner, Likhitha Surapaneni, Kyösti Sutinen, Michal Szpak, Francesca Floriana Tricomi, David Urbina-Gómez, Andres Veidenberg, Thomas A Walsh, Brandon Walts, Elizabeth Wass, Natalie Willhoft, Jamie Allen, Jorge Alvarez-Jarreta, Marc Chakiachvili, Bethany Flint, Stefano Giorgetti, Leanne Haggerty, Garth R Ilsley, Jane E Loveland, Benjamin Moore, Jonathan M Mudge, John Tate, David Thybert, Stephen J Trevanion, Andrea Winterbottom, Adam Frankish, Sarah E Hunt, Magali Ruffier, Fiona Cunningham, Sarah Dyer, Robert D Finn, Kevin L Howe, Peter W Harrison, Andrew D Yates, Paul Flicek

**Affiliations:** European Molecular Biology Laboratory, European Bioinformatics Institute, Wellcome Genome Campus, Hinxton, CB10 1SD, Cambridge, UK; European Molecular Biology Laboratory, European Bioinformatics Institute, Wellcome Genome Campus, Hinxton, CB10 1SD, Cambridge, UK; European Molecular Biology Laboratory, European Bioinformatics Institute, Wellcome Genome Campus, Hinxton, CB10 1SD, Cambridge, UK; European Molecular Biology Laboratory, European Bioinformatics Institute, Wellcome Genome Campus, Hinxton, CB10 1SD, Cambridge, UK; European Molecular Biology Laboratory, European Bioinformatics Institute, Wellcome Genome Campus, Hinxton, CB10 1SD, Cambridge, UK; European Molecular Biology Laboratory, European Bioinformatics Institute, Wellcome Genome Campus, Hinxton, CB10 1SD, Cambridge, UK; European Molecular Biology Laboratory, European Bioinformatics Institute, Wellcome Genome Campus, Hinxton, CB10 1SD, Cambridge, UK; European Molecular Biology Laboratory, European Bioinformatics Institute, Wellcome Genome Campus, Hinxton, CB10 1SD, Cambridge, UK; European Molecular Biology Laboratory, European Bioinformatics Institute, Wellcome Genome Campus, Hinxton, CB10 1SD, Cambridge, UK; European Molecular Biology Laboratory, European Bioinformatics Institute, Wellcome Genome Campus, Hinxton, CB10 1SD, Cambridge, UK; European Molecular Biology Laboratory, European Bioinformatics Institute, Wellcome Genome Campus, Hinxton, CB10 1SD, Cambridge, UK; European Molecular Biology Laboratory, European Bioinformatics Institute, Wellcome Genome Campus, Hinxton, CB10 1SD, Cambridge, UK; European Molecular Biology Laboratory, European Bioinformatics Institute, Wellcome Genome Campus, Hinxton, CB10 1SD, Cambridge, UK; European Molecular Biology Laboratory, European Bioinformatics Institute, Wellcome Genome Campus, Hinxton, CB10 1SD, Cambridge, UK; European Molecular Biology Laboratory, European Bioinformatics Institute, Wellcome Genome Campus, Hinxton, CB10 1SD, Cambridge, UK; European Molecular Biology Laboratory, European Bioinformatics Institute, Wellcome Genome Campus, Hinxton, CB10 1SD, Cambridge, UK; European Molecular Biology Laboratory, European Bioinformatics Institute, Wellcome Genome Campus, Hinxton, CB10 1SD, Cambridge, UK; European Molecular Biology Laboratory, European Bioinformatics Institute, Wellcome Genome Campus, Hinxton, CB10 1SD, Cambridge, UK; European Molecular Biology Laboratory, European Bioinformatics Institute, Wellcome Genome Campus, Hinxton, CB10 1SD, Cambridge, UK; European Molecular Biology Laboratory, European Bioinformatics Institute, Wellcome Genome Campus, Hinxton, CB10 1SD, Cambridge, UK; European Molecular Biology Laboratory, European Bioinformatics Institute, Wellcome Genome Campus, Hinxton, CB10 1SD, Cambridge, UK; European Molecular Biology Laboratory, European Bioinformatics Institute, Wellcome Genome Campus, Hinxton, CB10 1SD, Cambridge, UK; European Molecular Biology Laboratory, European Bioinformatics Institute, Wellcome Genome Campus, Hinxton, CB10 1SD, Cambridge, UK; European Molecular Biology Laboratory, European Bioinformatics Institute, Wellcome Genome Campus, Hinxton, CB10 1SD, Cambridge, UK; European Molecular Biology Laboratory, European Bioinformatics Institute, Wellcome Genome Campus, Hinxton, CB10 1SD, Cambridge, UK; European Molecular Biology Laboratory, European Bioinformatics Institute, Wellcome Genome Campus, Hinxton, CB10 1SD, Cambridge, UK; European Molecular Biology Laboratory, European Bioinformatics Institute, Wellcome Genome Campus, Hinxton, CB10 1SD, Cambridge, UK; European Molecular Biology Laboratory, European Bioinformatics Institute, Wellcome Genome Campus, Hinxton, CB10 1SD, Cambridge, UK; European Molecular Biology Laboratory, European Bioinformatics Institute, Wellcome Genome Campus, Hinxton, CB10 1SD, Cambridge, UK; European Molecular Biology Laboratory, European Bioinformatics Institute, Wellcome Genome Campus, Hinxton, CB10 1SD, Cambridge, UK; European Molecular Biology Laboratory, European Bioinformatics Institute, Wellcome Genome Campus, Hinxton, CB10 1SD, Cambridge, UK; European Molecular Biology Laboratory, European Bioinformatics Institute, Wellcome Genome Campus, Hinxton, CB10 1SD, Cambridge, UK; European Molecular Biology Laboratory, European Bioinformatics Institute, Wellcome Genome Campus, Hinxton, CB10 1SD, Cambridge, UK; European Molecular Biology Laboratory, European Bioinformatics Institute, Wellcome Genome Campus, Hinxton, CB10 1SD, Cambridge, UK; European Molecular Biology Laboratory, European Bioinformatics Institute, Wellcome Genome Campus, Hinxton, CB10 1SD, Cambridge, UK; European Molecular Biology Laboratory, European Bioinformatics Institute, Wellcome Genome Campus, Hinxton, CB10 1SD, Cambridge, UK; European Molecular Biology Laboratory, European Bioinformatics Institute, Wellcome Genome Campus, Hinxton, CB10 1SD, Cambridge, UK; European Molecular Biology Laboratory, European Bioinformatics Institute, Wellcome Genome Campus, Hinxton, CB10 1SD, Cambridge, UK; European Molecular Biology Laboratory, European Bioinformatics Institute, Wellcome Genome Campus, Hinxton, CB10 1SD, Cambridge, UK; European Molecular Biology Laboratory, European Bioinformatics Institute, Wellcome Genome Campus, Hinxton, CB10 1SD, Cambridge, UK; European Molecular Biology Laboratory, European Bioinformatics Institute, Wellcome Genome Campus, Hinxton, CB10 1SD, Cambridge, UK; European Molecular Biology Laboratory, European Bioinformatics Institute, Wellcome Genome Campus, Hinxton, CB10 1SD, Cambridge, UK; European Molecular Biology Laboratory, European Bioinformatics Institute, Wellcome Genome Campus, Hinxton, CB10 1SD, Cambridge, UK; European Molecular Biology Laboratory, European Bioinformatics Institute, Wellcome Genome Campus, Hinxton, CB10 1SD, Cambridge, UK; European Molecular Biology Laboratory, European Bioinformatics Institute, Wellcome Genome Campus, Hinxton, CB10 1SD, Cambridge, UK; European Molecular Biology Laboratory, European Bioinformatics Institute, Wellcome Genome Campus, Hinxton, CB10 1SD, Cambridge, UK; European Molecular Biology Laboratory, European Bioinformatics Institute, Wellcome Genome Campus, Hinxton, CB10 1SD, Cambridge, UK; European Molecular Biology Laboratory, European Bioinformatics Institute, Wellcome Genome Campus, Hinxton, CB10 1SD, Cambridge, UK; European Molecular Biology Laboratory, European Bioinformatics Institute, Wellcome Genome Campus, Hinxton, CB10 1SD, Cambridge, UK; European Molecular Biology Laboratory, European Bioinformatics Institute, Wellcome Genome Campus, Hinxton, CB10 1SD, Cambridge, UK; European Molecular Biology Laboratory, European Bioinformatics Institute, Wellcome Genome Campus, Hinxton, CB10 1SD, Cambridge, UK; European Molecular Biology Laboratory, European Bioinformatics Institute, Wellcome Genome Campus, Hinxton, CB10 1SD, Cambridge, UK; European Molecular Biology Laboratory, European Bioinformatics Institute, Wellcome Genome Campus, Hinxton, CB10 1SD, Cambridge, UK; European Molecular Biology Laboratory, European Bioinformatics Institute, Wellcome Genome Campus, Hinxton, CB10 1SD, Cambridge, UK; European Molecular Biology Laboratory, European Bioinformatics Institute, Wellcome Genome Campus, Hinxton, CB10 1SD, Cambridge, UK; European Molecular Biology Laboratory, European Bioinformatics Institute, Wellcome Genome Campus, Hinxton, CB10 1SD, Cambridge, UK; European Molecular Biology Laboratory, European Bioinformatics Institute, Wellcome Genome Campus, Hinxton, CB10 1SD, Cambridge, UK; European Molecular Biology Laboratory, European Bioinformatics Institute, Wellcome Genome Campus, Hinxton, CB10 1SD, Cambridge, UK; European Molecular Biology Laboratory, European Bioinformatics Institute, Wellcome Genome Campus, Hinxton, CB10 1SD, Cambridge, UK; European Molecular Biology Laboratory, European Bioinformatics Institute, Wellcome Genome Campus, Hinxton, CB10 1SD, Cambridge, UK; European Molecular Biology Laboratory, European Bioinformatics Institute, Wellcome Genome Campus, Hinxton, CB10 1SD, Cambridge, UK; European Molecular Biology Laboratory, European Bioinformatics Institute, Wellcome Genome Campus, Hinxton, CB10 1SD, Cambridge, UK; European Molecular Biology Laboratory, European Bioinformatics Institute, Wellcome Genome Campus, Hinxton, CB10 1SD, Cambridge, UK; European Molecular Biology Laboratory, European Bioinformatics Institute, Wellcome Genome Campus, Hinxton, CB10 1SD, Cambridge, UK; European Molecular Biology Laboratory, European Bioinformatics Institute, Wellcome Genome Campus, Hinxton, CB10 1SD, Cambridge, UK; European Molecular Biology Laboratory, European Bioinformatics Institute, Wellcome Genome Campus, Hinxton, CB10 1SD, Cambridge, UK; European Molecular Biology Laboratory, European Bioinformatics Institute, Wellcome Genome Campus, Hinxton, CB10 1SD, Cambridge, UK; European Molecular Biology Laboratory, European Bioinformatics Institute, Wellcome Genome Campus, Hinxton, CB10 1SD, Cambridge, UK; European Molecular Biology Laboratory, European Bioinformatics Institute, Wellcome Genome Campus, Hinxton, CB10 1SD, Cambridge, UK; European Molecular Biology Laboratory, European Bioinformatics Institute, Wellcome Genome Campus, Hinxton, CB10 1SD, Cambridge, UK; European Molecular Biology Laboratory, European Bioinformatics Institute, Wellcome Genome Campus, Hinxton, CB10 1SD, Cambridge, UK; European Molecular Biology Laboratory, European Bioinformatics Institute, Wellcome Genome Campus, Hinxton, CB10 1SD, Cambridge, UK; European Molecular Biology Laboratory, European Bioinformatics Institute, Wellcome Genome Campus, Hinxton, CB10 1SD, Cambridge, UK; European Molecular Biology Laboratory, European Bioinformatics Institute, Wellcome Genome Campus, Hinxton, CB10 1SD, Cambridge, UK; European Molecular Biology Laboratory, European Bioinformatics Institute, Wellcome Genome Campus, Hinxton, CB10 1SD, Cambridge, UK; European Molecular Biology Laboratory, European Bioinformatics Institute, Wellcome Genome Campus, Hinxton, CB10 1SD, Cambridge, UK; European Molecular Biology Laboratory, European Bioinformatics Institute, Wellcome Genome Campus, Hinxton, CB10 1SD, Cambridge, UK; European Molecular Biology Laboratory, European Bioinformatics Institute, Wellcome Genome Campus, Hinxton, CB10 1SD, Cambridge, UK; European Molecular Biology Laboratory, European Bioinformatics Institute, Wellcome Genome Campus, Hinxton, CB10 1SD, Cambridge, UK; European Molecular Biology Laboratory, European Bioinformatics Institute, Wellcome Genome Campus, Hinxton, CB10 1SD, Cambridge, UK; European Molecular Biology Laboratory, European Bioinformatics Institute, Wellcome Genome Campus, Hinxton, CB10 1SD, Cambridge, UK; European Molecular Biology Laboratory, European Bioinformatics Institute, Wellcome Genome Campus, Hinxton, CB10 1SD, Cambridge, UK; European Molecular Biology Laboratory, European Bioinformatics Institute, Wellcome Genome Campus, Hinxton, CB10 1SD, Cambridge, UK; European Molecular Biology Laboratory, European Bioinformatics Institute, Wellcome Genome Campus, Hinxton, CB10 1SD, Cambridge, UK; European Molecular Biology Laboratory, European Bioinformatics Institute, Wellcome Genome Campus, Hinxton, CB10 1SD, Cambridge, UK; European Molecular Biology Laboratory, European Bioinformatics Institute, Wellcome Genome Campus, Hinxton, CB10 1SD, Cambridge, UK; European Molecular Biology Laboratory, European Bioinformatics Institute, Wellcome Genome Campus, Hinxton, CB10 1SD, Cambridge, UK; European Molecular Biology Laboratory, European Bioinformatics Institute, Wellcome Genome Campus, Hinxton, CB10 1SD, Cambridge, UK; European Molecular Biology Laboratory, European Bioinformatics Institute, Wellcome Genome Campus, Hinxton, CB10 1SD, Cambridge, UK; European Molecular Biology Laboratory, European Bioinformatics Institute, Wellcome Genome Campus, Hinxton, CB10 1SD, Cambridge, UK; European Molecular Biology Laboratory, European Bioinformatics Institute, Wellcome Genome Campus, Hinxton, CB10 1SD, Cambridge, UK; European Molecular Biology Laboratory, European Bioinformatics Institute, Wellcome Genome Campus, Hinxton, CB10 1SD, Cambridge, UK; European Molecular Biology Laboratory, European Bioinformatics Institute, Wellcome Genome Campus, Hinxton, CB10 1SD, Cambridge, UK; European Molecular Biology Laboratory, European Bioinformatics Institute, Wellcome Genome Campus, Hinxton, CB10 1SD, Cambridge, UK; European Molecular Biology Laboratory, European Bioinformatics Institute, Wellcome Genome Campus, Hinxton, CB10 1SD, Cambridge, UK; European Molecular Biology Laboratory, European Bioinformatics Institute, Wellcome Genome Campus, Hinxton, CB10 1SD, Cambridge, UK

## Abstract

Ensembl (https://www.ensembl.org) has produced high-quality genomic resources for vertebrates and model organisms for more than twenty years. During that time, our resources, services and tools have continually evolved in line with both the publicly available genome data and the downstream research and applications that utilise the Ensembl platform. In recent years we have witnessed a dramatic shift in the genomic landscape. There has been a large increase in the number of high-quality reference genomes through global biodiversity initiatives. In parallel, there have been major advances towards pangenome representations of higher species, where many alternative genome assemblies representing different breeds, cultivars, strains and haplotypes are now available. In order to support these efforts and accelerate downstream research, it is our goal at Ensembl to create high-quality annotations, tools and services for species across the tree of life. Here, we report our resources for popular reference genomes, the dramatic growth of our annotations (including haplotypes from the first human pangenome graphs), updates to the Ensembl Variant Effect Predictor (VEP), interactive protein structure predictions from AlphaFold DB, and the beta release of our new website.

## INTRODUCTION

For over 20 years, Ensembl has been at the forefront of creating high-quality genome annotations, including genes, variants, regulatory regions and comparative genomics resources. We enable free access to the data in a variety of ways, from visualisations in the genome browser, programmatic access through the Application Programming Interfaces (APIs), querying via our tools and services, and downloading data through our FTP site.

Over the last two decades, the field of genomics has progressed from sequencing and assembling a handful of draft-quality eukaryotic genomes to the thousands available today. There has been a dramatic improvement in the protocols and technology used to sequence and assemble genomes (1), and in tandem the costs and expertise required to build a high-quality genome assembly have been greatly reduced. This has led to an unprecedented number of available genomes, better capturing genomic variation both within and across species (1–6).

Nowhere is this more evident than the human genome itself, with the recent release of the CHM13 genome assembly from the Telomere to Telomere Consortium (T2T) (6), marking the first essentially complete human genome. This has quickly been followed by 94 high-quality haplotype assemblies from 47 individuals, represented in the first large scale human pangenome graph, released as part of the Human Pangenome Reference Consortium (HPRC) (7). These efforts are broadening the representation of human haplotypes and mark an important step toward a more inclusive and diverse era of human genomics.

Species with large research communities continue to both regularly improve the quality of reference genomes, while also increasing the number of alternative assemblies per species. This is particularly evident in the livestock community where there are many examples of high-quality reference genomes and breed-specific genome assemblies (2,4,8–10), both of which are key to advancing breeding programs, which in turn enhance global food security (11–14).

While there is a trend of deeper representation of genomic variation within species, there is simultaneously an explosion of the breadth of eukaryotic genomes through the many global biodiversity efforts currently underway, which includes Darwin Tree of Life (DToL), the Vertebrate Genomes Project (VGP) and the Earth BioGenome Project (1,3,5). These initiatives have already led to hundreds of high-quality reference genomes across the eukaryotic tree, with almost 400 public reference genomes available from DToL alone. This will undoubtedly lead to a much clearer understanding of eukaryotic life and how genomes and their underlying functional elements evolve, in addition to helping with efforts to conserve global biodiversity.

Ensembl provides high-quality, consistent genomic analyses. These range from the annotation of repeats, to genes and gene trees, whole genome alignments, variants and regulatory features. Over the past year we have more than doubled our available genome annotations, with new data coming out as frequently as every two weeks through our Rapid Release website. We have adopted a strategy to ensure support for a rich variety of data types for key species, while continuing to expand our annotations across the tree of life.

In this manuscript we will highlight some of the resources added to the Ensembl platform over the past year and detail future directions.

### Deep resources for key species

Increasing the quality and diversity of resources available for popular species, with rich data and large communities (such as human, livestock and model species), is a key part of our mission. The past year has witnessed some major advances in genomics, including the first essentially complete human genome, the first draft human pangenome graph and large-scale availability of protein structure predictions. Below we describe our ongoing efforts to ensure that our annotation and resources evolve in tandem with these advances. These updates are available from www.ensembl.org (as of Ensembl release 108), with the exception of the human pangenome data, which are hosted on rapid.ensembl.org.

#### Human reference resources

The Ensembl/GENCODE human reference annotation (15) is used globally by major projects and consortia including the NHGRI-EBI GWAS Catalog (16), UK BioBank (17), ICGC/TCGA (18), gnomAD (19) and the Human Cell Atlas (20), which demands that the annotations reflect the latest scientific understanding and data.

Our team of manual annotation experts have worked on refining existing gene structures, and the addition of alternate transcripts and new loci. A total of 313 new and 1,918 updated lncRNA annotations have been added through the TAGENE process (15). In addition, there are 48 new and 1286 updated annotations for protein-coding loci. We have removed or deprecated low-quality genes or those that exist due to errors in the GRCh38 assembly; for example, we have reclassified 29 genes on chromosome 21 under the ‘Artifact’ biotype, as the recent released CHM13 assembly demonstrated the corresponding genes reside on an artificial duplication on the reference genome.

We have continued to work on the Matched Annotation from NCBI and EMBL-EBI (MANE) project (21) to increase the consistency of annotation between the Ensembl/GENCODE and NCBI’s RefSeq (22) gene sets. A total of 19 062 (99.1%) of human protein-coding genes now have an agreed representative transcript. This ensures consistency on the agreed default transcript for each protein-coding gene, while also resolving differences between the reported transcript sequence and structure in each resource.

The Ensembl human variation resources have been updated to include a further 13 million short variants (714 million total), 1 million structural variants (6 million total), and 1 million phenotype associations (16 million total). We have added population frequency data from gnomAD version 3.1.2, which spans 76 156 genomes of diverse ancestry and created tracks for key Human Genome Structural Variation studies.

The Ensembl Regulatory Build (23) now consists of 622 461 regulatory features across 118 epigenomes. We have increased the strictness of our motif feature annotation algorithm and now only display motif features that overlap a ChIP-seq peak in at least one cell type. This increases the confidence that the reported motifs have biological significance. We removed the standalone promoter-flanking feature type, as these are not strictly adjacent to promoters, and instead relabelled these regions as enhancers, which better reflects how they are identified. Additionally, we have made minor updates to the display of regulatory features, ensuring that features of the same type are displayed on the same row. Taken together these changes both improve the confidence in our regulatory region annotation and ensure more consistent categorisation and display.

**Figure 1:**
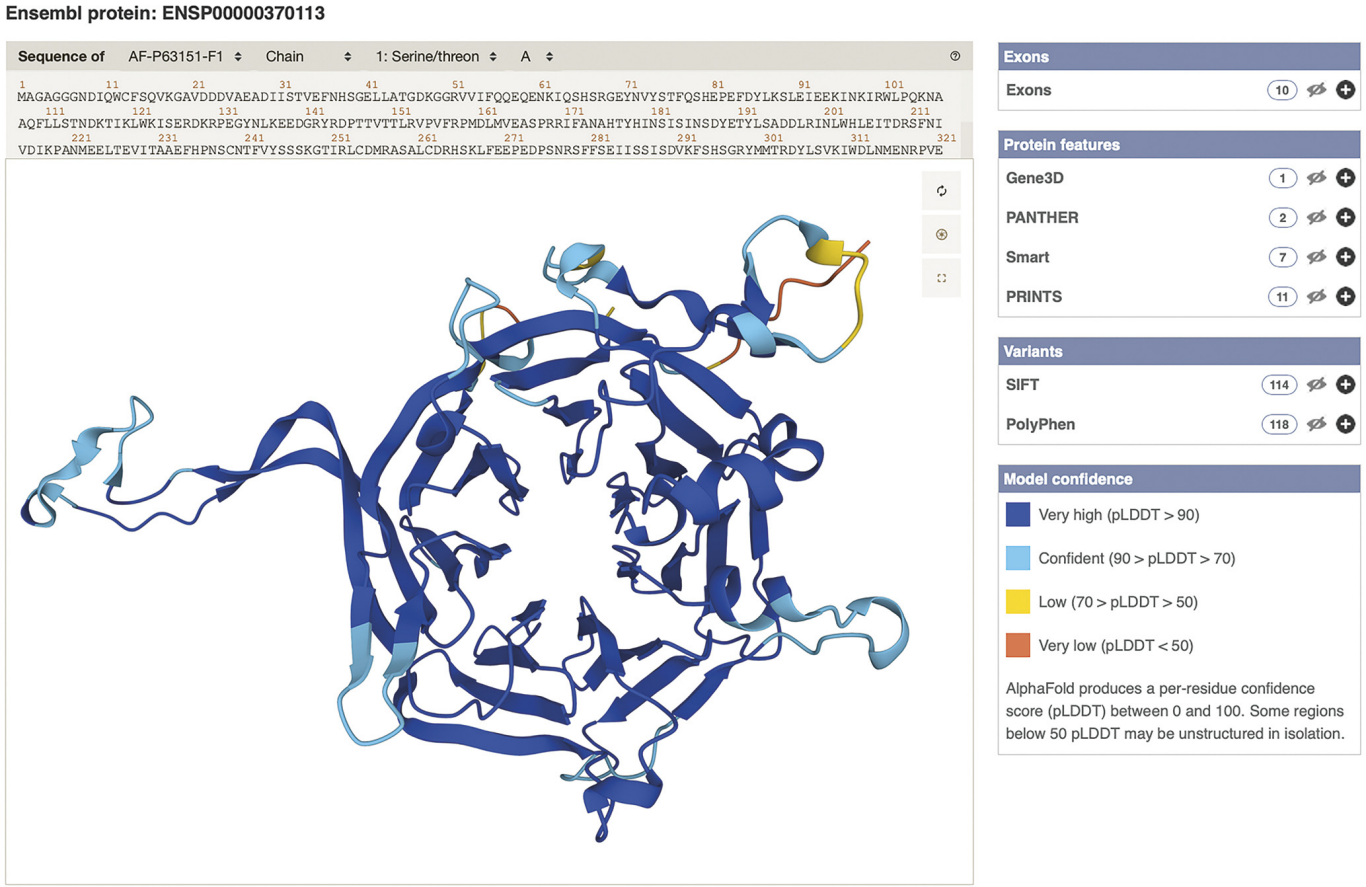
AlphaFold structure prediction for the product of PPP2R2A. AlphaFold structures can be viewed through our new browser widget, via the ‘AlphaFold predicted model’ link in the left hand menu of any transcript page. Note that AlphaFold structures are currently limited to the primary coding transcript of each protein-coding gene, usually the one listed at the top of the transcript table.

With the advent of AlphaFold2 (24), protein structure predictions are available for almost every human protein-coding gene. Structures from AlphaFold DB (25) for 19 013 human protein-coding genes are now viewable through our new AlphaFold browser widget, accessible via the ‘AlphaFold predicted model’ link in the left-hand menu of any transcript page. For transcripts where a model is available, it is possible to highlight individual residues, variants, exon boundaries, and protein features on the predicted structure. This includes the ability to focus on one feature at a time, for example highlighting a single exon. By default, the structure is colour coded based on a pLDDT score, to show the confidence of the predictions (Figure 1).

#### Towards an annotated human pangenome

The HPRC has generated the first draft human pangenome graphs, which contain the maternal and paternal haplotypes for 47 individuals, along with the CHM13 T2T assembly and the GRCh38 reference assembly.

As part of these efforts, we have generated gene annotation for each of the 95 haplotypes, including the CHM13v2.0 assembly, through a process of mapping the Ensembl/GENCODE reference gene set (version 38) from GRCh38 to each haplotype, including the annotation of new loci arising from copy number variation (7). Due to the high-quality nature of the underlying sequences, high mapping percentages across the haplotypes are observed, with the largest variability coming from pseudogenes. We have additionally lifted over variation data from gnomAD Genomes (version 3.1.2) and ClinVar (version 2022-07-09) for an initial 89 haplotypes, including CHM13 T2T. To facilitate the annotation of variants called against the new assemblies, these data are available through our FTP site in VCF alongside Ensembl VEP caches containing the remapped Ensembl/GENCODE gene set. The underlying whole genome alignment graphs, generated by UCSC using Cactus (26), are also available through our FTP site.

We have released initial annotation sets for each of these genomes through our Rapid Release site and built a dedicated HPRC project page (https://projects.ensembl.org/hprc). We will regularly update this site with new haplotypes as they become available and will update the gene annotation for all assemblies to reflect the latest Ensembl/GENCODE data and utilise enhanced mapping methods.

#### Expanded support for other popular species

In collaboration with UniProt (27), our mouse Ensembl/GENCODE gene set has been refined, through the Genome Integration with FuncTion and Sequence (GIFTS, https://www.ebi.ac.uk/gifts/) project. GIFTS has helped improve consistency between protein products present in UniProt and the representation of the corresponding transcript structures on the genome. When the two are in conflict, annotators and curators have determined the cause and then implemented a change to either the genome annotation or the protein record (or both). A total of 104 protein-coding genes have been updated. The mouse lncRNA set has been significantly improved through TAGENE, with 1388 new and 1340 updated annotations.

We have improved and expanded our chicken and pig resources to incorporate tissue-specific developmental data generated as part of the GENE-SWitCH project (https://www.gene-switch.eu/), that is part of the EuroFAANG initiative (https://eurofaang.eu/). For chicken, we have updated the reference genome from the wild Jungle Fowl to a Broiler breed and also added the White Leghorn breed, both of which are commercially important. All three genomes have been annotated utilising the GENE-SWitCH data along with other publicly available transcriptomic data. For the Broiler reference, we display allele frequency data from the European Variation Archive (28) and have also generated ATAC-seq tracks for Broiler and Jungle Fowl. For pig, we have updated the Duroc reference breed annotation using developmental transcriptomic data and display new ATAC-seq tracks.

For fish we have new, high-quality genome assemblies and annotations for Atlantic salmon, common carp and European seabass, produced as part of the EuroFAANG AQUA-FAANG project (https://www.aqua-faang.eu/). We have updated our rainbow trout reference assembly and annotation and added AlphaFold predictions for Zebrafish proteins.

### Annotating the eukaryotic tree of life

Below we describe our work supporting global biodiversity efforts. The data can be accessed via our Rapid Release website.

#### Efficient, high-quality genome annotation

**Figure 2:**
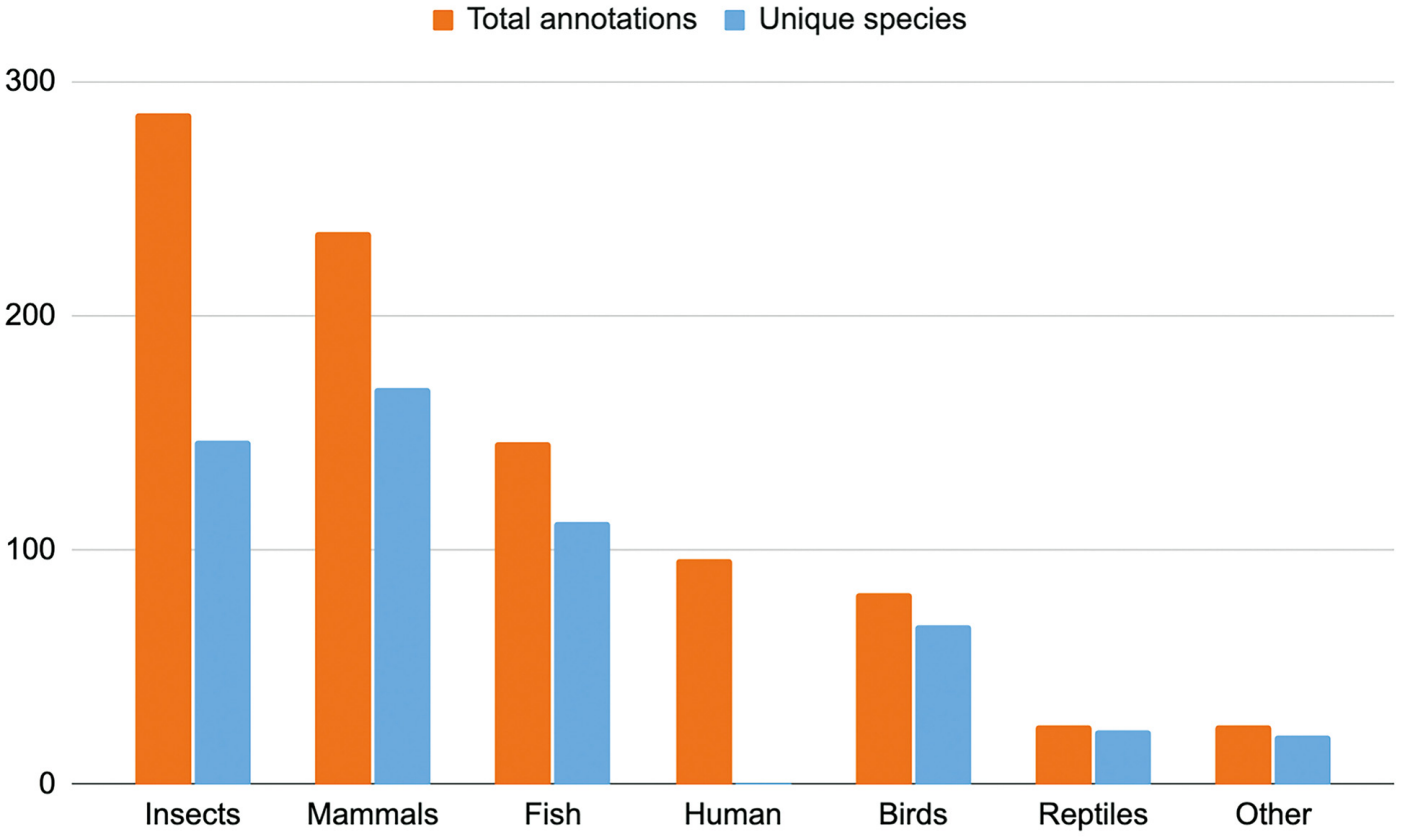
Total annotations and species offered through Ensembl. This represents total annotations across all species where we have generated annotations through the Ensembl Annotation System (as of October 2022). Human has been given a separate category to reflect the large number of annotations generated as part of the HPRC.

Over the past twelve months we have released our largest ever number of annotated assemblies (see Figure 2), with over 570 new annotations. The majority of these are in support of biodiversity, driven primarily by genomes generated as part of DToL. We have not only annotated the primary haplotypes of these species, but also the alternative haplotypes wherever a high-quality alternative haplotype is present. These data are released approximately every two weeks via our Rapid Release website.

Traditionally, we have focused on annotating vertebrate genomes (still accounting for around 50 percent of our current total annotations), but we are rapidly expanding our analysis across several non-vertebrate groups. We have significantly improved our available resources for insects, releasing a total of 286 assemblies across 147 species. Lepidoptera (butterflies and moths) account for the bulk of these genomes and we have also extensively annotated the genus Bombus, with 26 species of bumble bee now available. Other annotations include species from Hymenoptera, Diptera, Coleoptera, a mollusc and a cnidarian.

To help assess the completeness of these annotations, which vary based on the amount of publicly available transcriptomic data and the suitability of the aligned proteins, we have started to generate BUSCO (29) scores for our annotations as standard. These have been made available through our Darwin Tree of Life project page (https://projects.ensembl.org/darwin-tree-of-life/), and in future will also be available directly on Rapid Release.

#### Rapid, draft annotation for species lacking transcriptomic data

The Ensembl annotation system uses transcriptomic data to build high-quality gene sets, but for many species there are no available transcriptomic data. This is particularly true of critically endangered species. Despite this, there is a need to support the communities studying these species with draft genome annotations. We have used the BRAKER2 (30) annotation tool to create draft annotations for species that do not yet have transcriptomic data. Protein data from both UniProt and OrthoDB (31) is used to help provide hints to guide BRAKER2. As of October 2022 there are 162 draft gene sets for 88 species of Lepidoptera using this approach.

#### Homologies and alignments at scale

The Ensembl homology pipeline identifies homologues using Diamond (32) to search against a set of 9 key eukaryotic species (namely human, chicken, zebrafish, *D. melanogaster*, *C. elegans, A. thaliana*, rice, *P. falciparum* and *S. cerevisiae*) and 29 other reference genomes selected based on the clade to which the target species belongs (33).

Every species available via the Rapid Release browser now has a set of homologues calculated for every protein-coding gene, with information on the type of homology (reciprocal best hit or best hit), the percent identity and coverage of the hit against the query and the gene name associated with the hit (if present). At time of writing this equates to over 108 million homologies.

The number of species included in our whole genome alignments have been expanded, with a new whole genome alignment of 38 fish species, generated as part of the AQUA-FAANG project. The alignment was built using Cactus and can be downloaded from the Rapid Release FTP site (https://ftp.ensembl.org/pub/rapid-release/data_files/multi/hal_files/).

#### Building an extensive collection of repeat libraries

Our collection of freely available repeat libraries (https://ftp.ebi.ac.uk/pub/databases/ensembl/repeats/unfiltered_repeatmodeler/species/) has increased the number of species represented by 54%.

**Figure 3:**
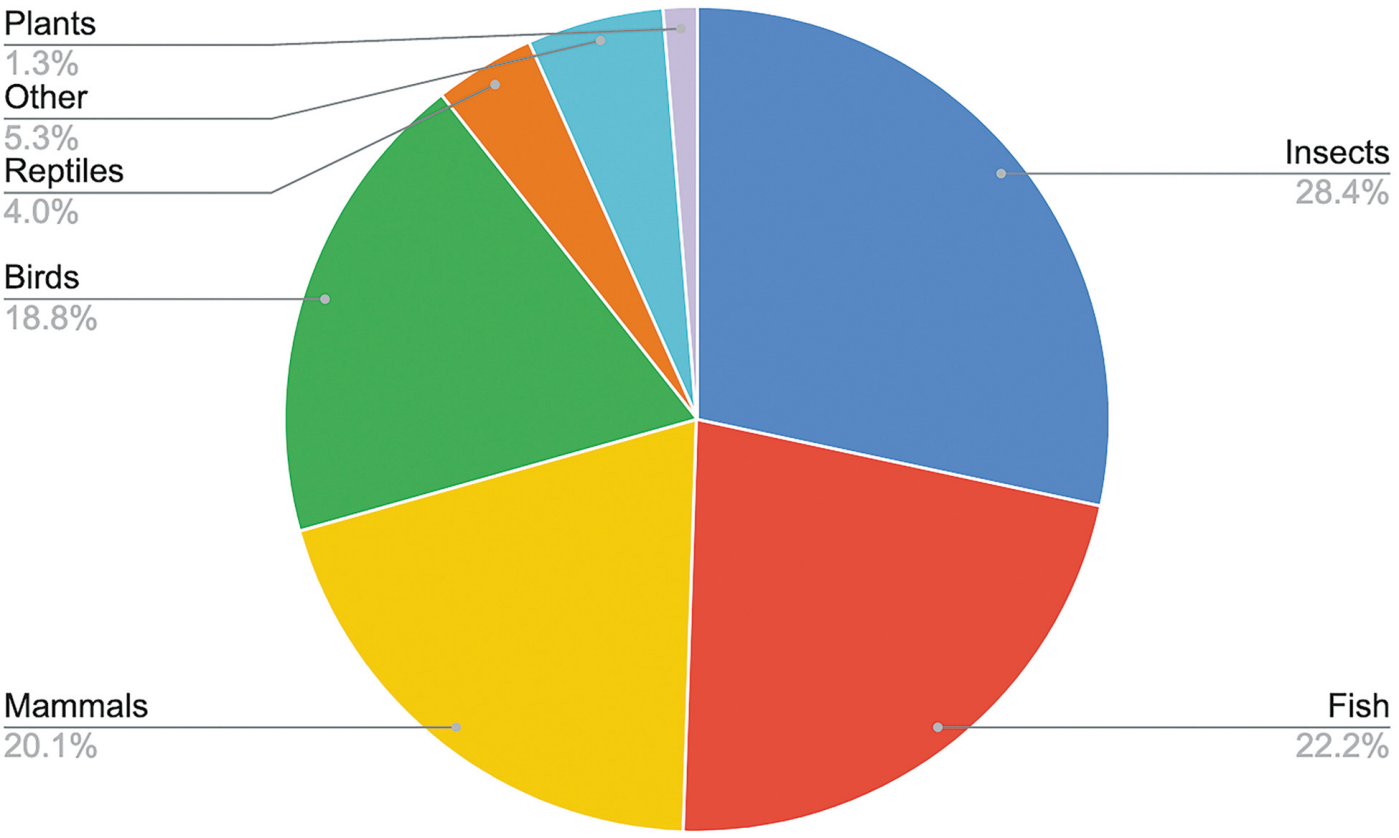
Distribution of eukaryotic repeat libraries. RepeatModeler-derived libraries are now available on our FTP site for over 1100 species. These libraries are released as soon as the RepeatModeler analysis has completed. The ‘Other’ category includes molluscs, protists, arthropods and vertebrates not falling into the clades defined above.

We distribute libraries for 2792 genome assemblies, across 1647 different species (as of October 2022), adding libraries for a further 579 species over the past year. To improve the quality of the libraries we updated to using the recently released version 2 of RepeatModeler (34). The libraries now cover a large number of non-vertebrate species, primarily insects, but also plants, protists and molluscs (Figure 3).

While the majority of libraries generated over the past twelve months represent newly sequenced species, we have continued to update libraries for existing species in the collection as newer, higher-quality genome assemblies become available, as in general newer assemblies have significantly better representation of repeats compared to older short-read assemblies (35).

### Empowering genome interpretation and analysis

In this section we describe updates to our new website, tools, services and training programs.

#### A beta release of our new website

The beta version of our new website has been released (https://beta.ensembl.org). This initial release represents an overview of some of the major design decisions behind the new browser. These include an app-based approach to functionality, a highly responsive genome browser, simplified displays and the ability to browse multiple species of interest simultaneously. Six key species have been included: human, *D. melanogaster*, *C. elegans*, wheat, *P. falciparum* and *E. coli*. For human, we have included both GRCh37 and GRCh38 assemblies, as a precursor to broader support for alternative assemblies in future iterations.

**Figure 4:**
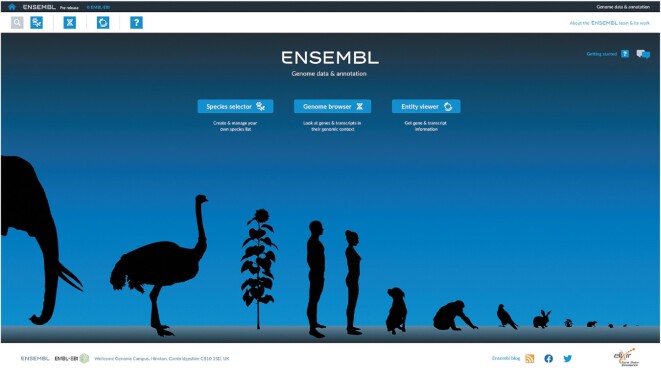
Homepage for the new Ensembl website. The homepage acts as a gateway for interacting with our data via the species selector, genome browser and entity viewer apps. The new website will eventually bring all species across the current set of Ensembl websites under a single infrastructure.

Initial functionality revolves around three apps: the species selector, the genome browser, and the entity viewer (Figure 4). The species selector allows the selection of one or several species (including alternate assemblies for a species), providing an overview of key metrics such as assembly contiguity and the number of features annotated. Selected species are then automatically carried into the genome browser and entity viewer. The new genome browser enables an intuitive overview of a genome, allowing rapid panning and zooming, from the level of viewing an entire chromosome right down to the underlying nucleotide sequence. The entity viewer displays a detailed view of a specific gene, from the exon structure of its transcripts to the function of the gene and resulting protein. The beta site also includes the ability to run BLAST (36) searches for multiple sequences against any of the seven currently available genomes, with results being presented in a straightforward tabular view.

#### Updates to the ensembl VEP

The Ensembl VEP (37) is a powerful toolset for variant annotation and prioritisation. We have continued to extend the available analysis options and have this year integrated predictions from EVE (38), which employs an evolutionary model of variant effect, and CAPICE (39), a machine-learning based method for prioritising pathogenic variants. Ensembl VEP also now reports when a variant which causes a premature stop codon results in a transcript likely to escape nonsense-mediated decay; such variants are thought to be relevant in Mendelian disease (40).

We have extended the protein annotations available in Ensembl VEP beyond the previous reporting when a variant falls in a protein domain and listing SwissProt matches and protein family information. The web interface now displays a variant's location on AlphaFold-predicted protein structures in an interactive visualisation. Variants lying in a protein location shown to affect protein interaction, as described in the IntAct database (41), are reported with links to the IntAct website and any journals describing the variant's effect for further information. To aid identification of potential disease-associated variants, Gene Ontology terms are also now reported, describing the function, the cellular location and the associated biological processes of the product of any gene a variant lies within.

#### Tracking transcripts through tark

Our transcript archive service, Tark (http://tark.ensembl.org/), allows extremely fine-grained tracking of changes to the annotation of the transcript sequences and structures from both Ensembl and RefSeq. Recent updates include the ability to track changes to the gene biotype (e.g. if a protein-coding gene was reclassified as a pseudogene). We have also added support for comparing the sequence and structure of GRCh37 annotations to MANE Select and MANE Plus Clinical transcripts annotated on GRCh38.

#### Outreach and training

During the course of the last year we delivered training, both in-person and virtual to 1805 participants from across the globe. In February 2022, we returned to in-person training, holding 3 in-person workshops, attended by over 200 participants. In addition, we ran a total of 46 virtual workshops, 20 of which were targeted at low and middle income countries including Colombia, India and Nigeria.

The training we deliver primarily covers our genome browser, but we also run more specialised training courses covering how to use our REST API, our variation resources and the Ensembl VEP. All training materials are made available via our training pages (https://training.ensembl.org/) where you can also find information about attending or hosting a workshop. You can also contact us via our helpdesk (helpdesk@ensembl.org).

## SUMMARY AND FUTURE DIRECTIONS

Over the past 12 months, we have released new genome annotations for key species, including the haplotypes from the draft human pangenome, updated our variant sets and regulatory regions, and imported protein structure predictions from AlphaFold DB. The number of genomes we have annotated has more than doubled to 829, expanding further across the eukaryotic tree of life. Our main website, www.ensembl.org, continues to focus on reference genome updates for vertebrate and model species, with high levels of data integration, including gene trees, multiple alignments and BioMart availability for the majority of species, and variants and regulatory data for select species. Our Rapid Release site has become the primary location for new species, particularly those arising from biodiversity initiatives, and also for accessing alternative haplotypes, breeds and strains. It continues to grow in terms of the resources available, with all species now having an associated set of homologies. The launch of our beta site points towards the future of our platform, and will eventually house all our species and annotations in one location, under an entirely new interface and infrastructure. It will retain the best elements of our resources from the previous two decades, while dealing with the shortcomings of our current infrastructure, particularly in terms of updating the look and feel, and dramatically improving the experience of browsing a genome.

Over the coming year, we expect a major increase in the number of pangenome initiatives for key species, particularly for model organisms and species related to agriculture. The number of haplotypes represented in the human pangenome will also continue to expand through the efforts of the HPRC and other initiatives. In addition to the biodiversity initiatives that are already well underway such as the VGP and DToL, we will see several new initiatives gather pace, including the Canadian BioGenome Project, African BioGenome Project and the European Reference Genome Atlas.

To ensure the value of these genomes are maximised, we will continue to release annotations, tools and services to allow researchers to ask increasingly complex questions. A particular focus will be on expanding our comparative resources, through the provision of gene trees and orthologue calls for all species via the integration of OrthoFinder (42) into our gene tree pipeline. We also plan to increase the number of Cactus alignments we provide to cover both more eukaryotic clades and include more species within each alignment. Similarly, as more variant and regulatory data become available, we will continue to broaden the types of data we produce across species. AlphaFold DB now includes predictions for over 200 million proteins from UniProt, as a result, we will expand our provision of AlphaFold models across all species where predictions are available.

We will continue to work towards our goal of providing freely accessible, high-quality data and tools across the tree of life.

## DATA AVAILABILITY

All Ensembl integrated data are available without restriction from the main website (https://www.ensembl.org), the Rapid Release site (https://rapid.ensembl.org), in bulk from the FTP site (https://ftp.ensembl.org) and programmatically via the REST API (https://rest.ensembl.org). Ensembl code is available from GitHub (https://github.com/Ensembl) under an open source Apache 2.0 licence. News about our releases and services can be found on our blog (https://www.ensembl.info), our announce mailing list (https://lists.ensembl.org/mailman/listinfo/announce), Twitter (@ensembl; https://twitter.com/ensembl) and Facebook (https://facebook.com/Ensembl.org). Ensembl and Ensembl VEP are registered trademarks of EMBL.
